# Case report: Whole exome sequencing reveals a novel splicing variant of *ANKRD17* gene in a Chinese male juvenile with developmental delay and transient tic disorder

**DOI:** 10.3389/fgene.2024.1422469

**Published:** 2024-09-09

**Authors:** Jing Chen, Shuo Yang, He Wang, Hongjing Wang, Yuanyuan Xiao, Shanling Liu

**Affiliations:** ^1^ Department of Medical Genetics/Prenatal Diagnostic Center, West China Second University Hospital, Sichuan University, Chengdu, China; ^2^ Key Laboratory of Birth Defects and Related Diseases of Women and Children (Sichuan University), Ministry of Education, Chengdu, China; ^3^ Department of Obstetrics and Gynecology, West China Second University Hospital, Sichuan University, Chengdu, China

**Keywords:** whole exome sequencing, Chopra-Amiel-Gordon syndrome, ANKRD17, mRNA analysis, splicing abnormality

## Abstract

**Background:**

The Ankyrin Repeat Domain Containing Protein 17 (*ANKRD17*, OMIM:615929) gene is a protein-coding gene associated with diseases such as Chopra-Amiel-Gordon Syndrome and Non-Specific Syndromic Intellectual Disability. The protein encoded by *ANKRD17* gene belongs to the ankyrin repeat-containing protein family, which is one of the most widely existing protein domains that exclusively mediate protein-protein interactions. To date, the research and reports on the *ANKRD17* gene are limited.

**Case presentation:**

Trio whole exome sequencing (Trio-WES) was conducted on the proband and his unaffected parents to elucidate the genetic etiology in the proband, who was clinically diagnosed with developmental delay and other phenotypes. Subsequently, Sanger sequencing was employed for validation of the identified candidate variant. Furthermore, RNA analysis was utilized to ascertain the impact of the variant on splicing. The WES revealed a novel heterozygous *ANKRD17* splicing variant (c.7248 + 1G>A) in the proband, but not detected in his unaffected parents. And the presence of the splicing variant of the *ANKRD17* gene was valided by the Sanger sequencing subsequently. And the RNA analysis confirmed that the novel variant was predicted to result in loss of donor splice site, and the analysis at mRNA level confirmed that it leads to exon 32 skipping (r.7100_7278del179) and causes premature termination of translation to the protein (p.D2357fs), therefore is classified as pathogenic.

**Conclusion:**

Our study reported a novel splicing variant in *ANKRD17* gene, which may be associated with partial eating, frequent urination, and tic syndrome. This finding expands both the phenotypic and genotypic spectrum of *ANKRD17* gene. Although there is currently no curative therapy available for *ANKRD17* gene variants, a definitive diagnosis of its genetic etiology is significant for genetic counseling and family planning purposes. Furthermore, this is the first reported case of the *ANKRD17* gene in China.

## Background

The *ANKRD17* gene, a downstream effector of cyclin E/CDK2, plays a pivotal role in cell cycle and DNA progression (Deng et al., 2009). The *ANKRD17* gene engages with molecules that facilitate immune responses towards bacteria and viruses. It also has been proposed that knockdown and overexpression of *ANKRD17* is functionally implicated in NOD2 and NOD1 mediated responses to bacteria in diverse human cell lines. Furthermore, the ankyrin repeat-containing protein, encoded by the *ANKRD17* gene, is one of the most widely existing protein domains that exclusively mediate protein-protein interactions. Additionally, this gene’s encoded protein participates in both DNA replication and various functions (Wang et al., 2012; Menning and Kufer, 2013; Li et al., 2006). The clinical phenotype associated with this gene is characterized by Chopra-Amiel-Gordon syndrome (MIM:619504). The major phenotypic characteristic of 34 individuals from 32 families with Chopra-Amiel-Gordon syndrome were developmental delay (variable), intellectual disability (variable), language develop delay, feeding difficulties, non-specific MRI and EEG abnormalities, seizures, a predisposition to recurrent infections, and others. Furthermore, a significant number of individuals exhibit similar facial dysmorphism (Chopra et al., 2021).

The infrequency of reports concerning the *ANKRD17* gene is notable. To date, only 30 damage mutation (DM) variants have been documented in the Human Gene Mutation Database (HGMD, http://www.hgmd.org), including 14 missense variants, two splice site variants, eight small deletions, two small insertions, two in-frame insertion-deletions, and one microdeletion (spanning 1.16 Mb). Except for cases in which parental samples could not be obtained, the other variants were *de novo*. In this study, we firstly report a novel splicing variant (c.7248 + 1 G>A) that leads to the skipping of exon 32 in the *ANKRD17* gene. Our findings not only expand the genotypic and phenotypic spectrum of *ANKRD17* gene but also provide valuable information for genetic counseling. Furthermore, this is the first case report of the *ANKRD17* gene in China.

### Case presentation

#### Clinical data

**FIGURE 1:**
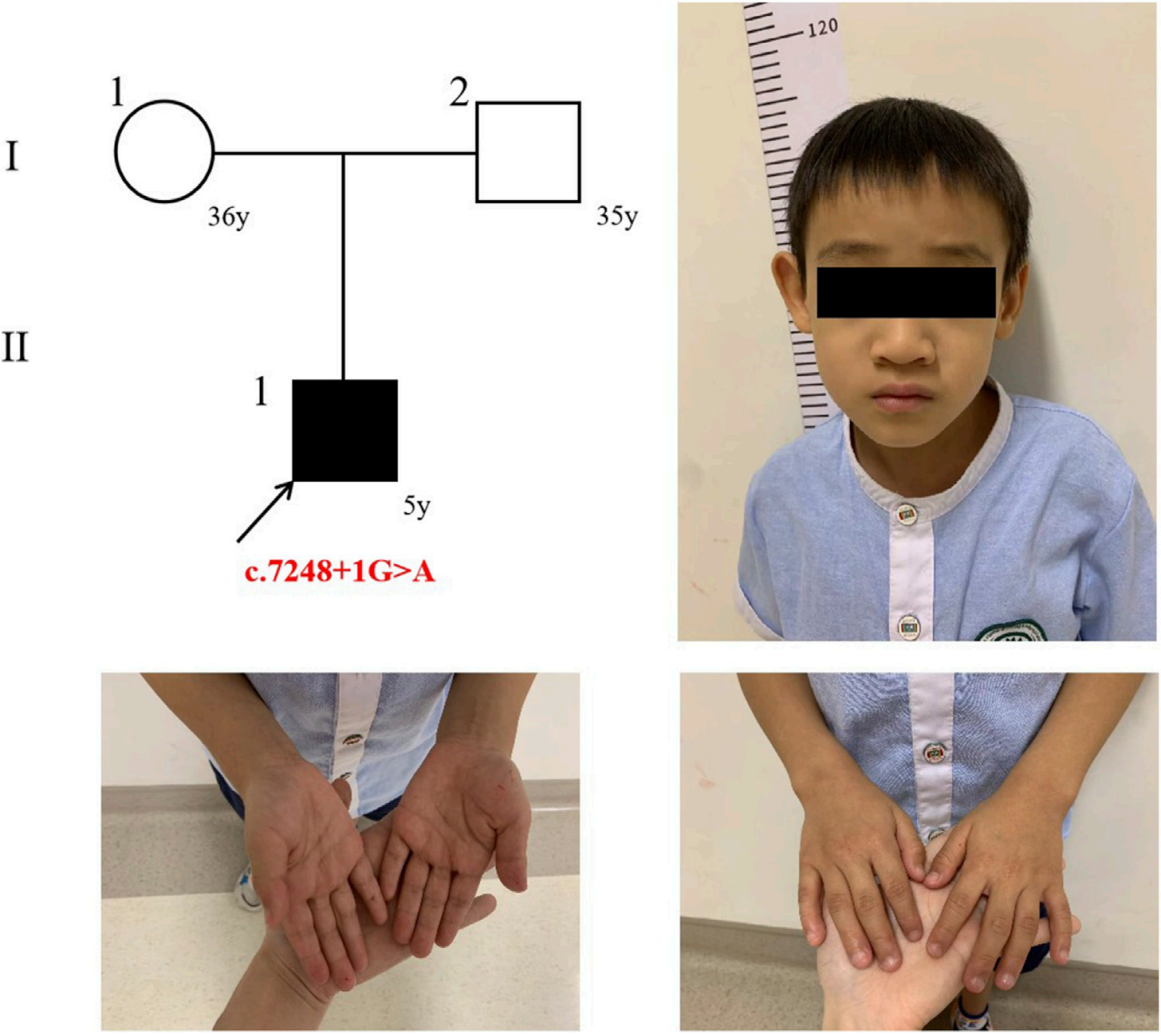
The trio’s pedigree and the proband’s photograph.

The proband is a 5-year-old male child. At the age of 2 years, the proband exhibited frequent blinking and involuntary laryngeal vocalization without apparent triggers. Additionally, the proband primarily demonstrated attention deficit and hyperactivity disorder (ADHD), mental and language retardation and feeding difficulty. No abnormalities were observed in the proband during the fetus or neonatal period. Detailed description of clinical manifestations was presented in Table 1 and Figure 1. This study was granted ethical approval by the Medical Ethics Committee of West China Second University Hospital, Sichuan University (Chengdu, China).

**TABLE 1:** The Clinical features of the proband.

Patient	Proband
Variant	NM_001286771.3,ANKRD17,c.7248 + 1G>A
Inheritance	*De Novo*
Current Age	5 years
Age of diagnosis	2^+^ years
Gender	Male
Weight,kg	16.5
Height,cm	109
Head Circumference,cm	49.5
Weeks Of Gestation	Term birth
Facial deformity	No obvious anomaly
Neurological Phenotype	
Intellectual Disability	Yes
Language development delay	Yes
Motor development	No obvious abnormality
Behavior Disorders	Intermittent blinking, Involuntary laryngeal vocalization; Attention Deficit and Hyperactivity
Assessment results	The total IQ score was 61 (95% confidence interval: 57–68), corresponding to a percentile grade of just 0.5%; Tic symptoms are moderate in severity; ADHD (combination type)
MRI Brain	Normal
Electroencephalogram	Normal
Miscellaneous	
Hearing loss	No
Feeding Difficulties	The subject exhibits an exclusive preference for plant-based foods, such that the consumption of animal products elicits aversive responses, including vomiting
Other anomaly	Recurrent pulmonary infections; Unexplained frequency of urination at 4 years, self-healing subsequently

Electroencephalogram:EEG, Attention deficit and hyperactivity disorder; ADHD, Intelligence quality:IQ.

#### Whole exome sequencing and analysis

The gDNA was extracted from the proband and parents’ (II1, I1 and I2) peripheral blood leukocytes using QIAamp DNA Blood Mini Kit (QIAGEN), following the manufacturer’s instruction. WES was performed using gDNA from II1, I1 and I2. Exome capture sequencing was performed using NanoWES Human Exome V1 (Berry genomics) according to the manufacturer’s protocol. The exon-enriched libraries were sequenced through Illunima NovaSeq6000 platform. Then, all reads were aligned to the reference human genome GRCh38/hg38 with Burrows-Wheeler Aligner (BWA). Then, singlenucleotide variants (SNVs) and small insertions or deletions (InDels) were identified by GATK Unified Genotyper (broadinstitute.org/). The ENLIVEN variants annotation interpretation system (Berry genomics) were used for functional annotation. High frequency variants (frequency over 5%) from public databases including GnomAD (http://gnomad.broadinstitute.org/) and 1000 Genomes Project (1000G) (http://browser.1000genomes.org) were excluded. Pathogenicity of SNVs was evaluated based on related scientific literature and disease databases, including ClinVar (http://www.ncbi.nlm.nih.gov/clinvar), PubMed (https://www.ncbi.nlm.nih.gov/pubmed/), OMIM (http://www.omim.org), HGMD (http://www.hgmd.org), etc. And the candidate pathogenic variants related to proband’s phenotypes were evaluated according to American Society for Medical Genetics and Genomics (ACMG) guidelines (Richards et al., 2015).Variations were classified into five categories according to the ACMG guidelines: pathogenic, likely pathogenic, uncertain significance, likely benign and benign. After ensuring data quality control for WES in this study, variation analysis was conducted according to the established process. Please refer to Supplemental Figure 1 for a detailed analysis flow chart.

#### Validation by Sanger sequencing and transcript analysis

The suspected splicing variant of the *ANKRD17* gene was validated by Sanger sequencing, and sequencing was performed using PCR primers designed with Primer Premier 5. Te sequences of the gDNA primers used was *ANKRD17*-F:5′-GGATAGGTGCTACTGGAGGAA-3′ and *ANKRD17*-R:5′-TGGAGCGAGATAGTACAGGAAT-3′. PCR products were sequenced using an ABI 3500 Genetic Analyser (Termo Fisher Scientifc) for *ANKRD17* c.7248 + 1G>A.

To figure out if the variant affects splicing, RNA was extracted from fresh peripheral blood of the trio (I1, I2, and II1) using the RNApure Blood Kit (CWBIO, CW0582S), according to the manufacturer’s instructions. Complementary DNA (cDNA) was then amplified using a primer set designed to amplify *ANKRD17* from exon 31 to exon 33; F:5′-GTAGGACATAGTGGCATCTG-3′, R:5′-ATAGGTGCTACTGGAGGAAT-3′. The PCR product were electrophoresed in 2% agarose gel, and bands extracted from agarose gel bands and subjected to Sanger sequencing.

## Results

**FIGURE 2:**
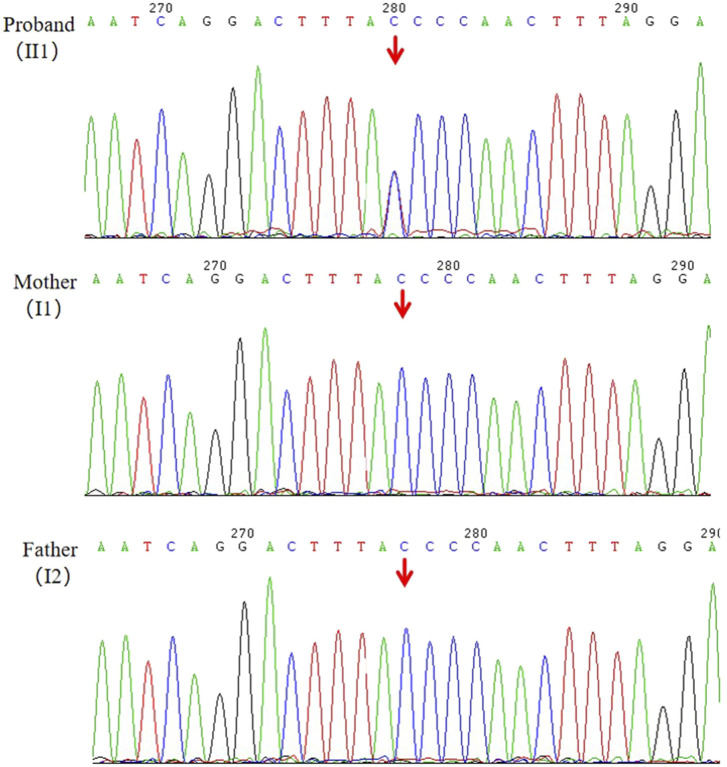
Validation of the candidate variant (*ANKRD17*,c.7248 + 1G>A) of gDNA (II1, I1, I2) by Sanger sequencing.

Following WES, we identified a novel heterozygous variant (NM_001286771.3, c.7248 + 1G>A) in the *ANKRD17* gene for the proband (II1), which was not present in his parents (I1, I2). The reference transcript of *ANKRD17* gene in the HGMD database is NM_001286771.3. The average sequencing depth of WES on the *ANKRD17* gene was 87×. The presence of the variant was further validated through Sanger sequencing (Figure 2). The proband, who was clinically diagnosed with DD and other clinical phenotypes, had the variant, while it was not detected in either the proband’s mother or father, as expected. Currently, this variant has not been reported in population-control databases, such as ExAC Browser, 1000 Genomes Project, gnomAD, or in disease databases, such as HGMD or ClinVar database.

**FIGURE 3:**
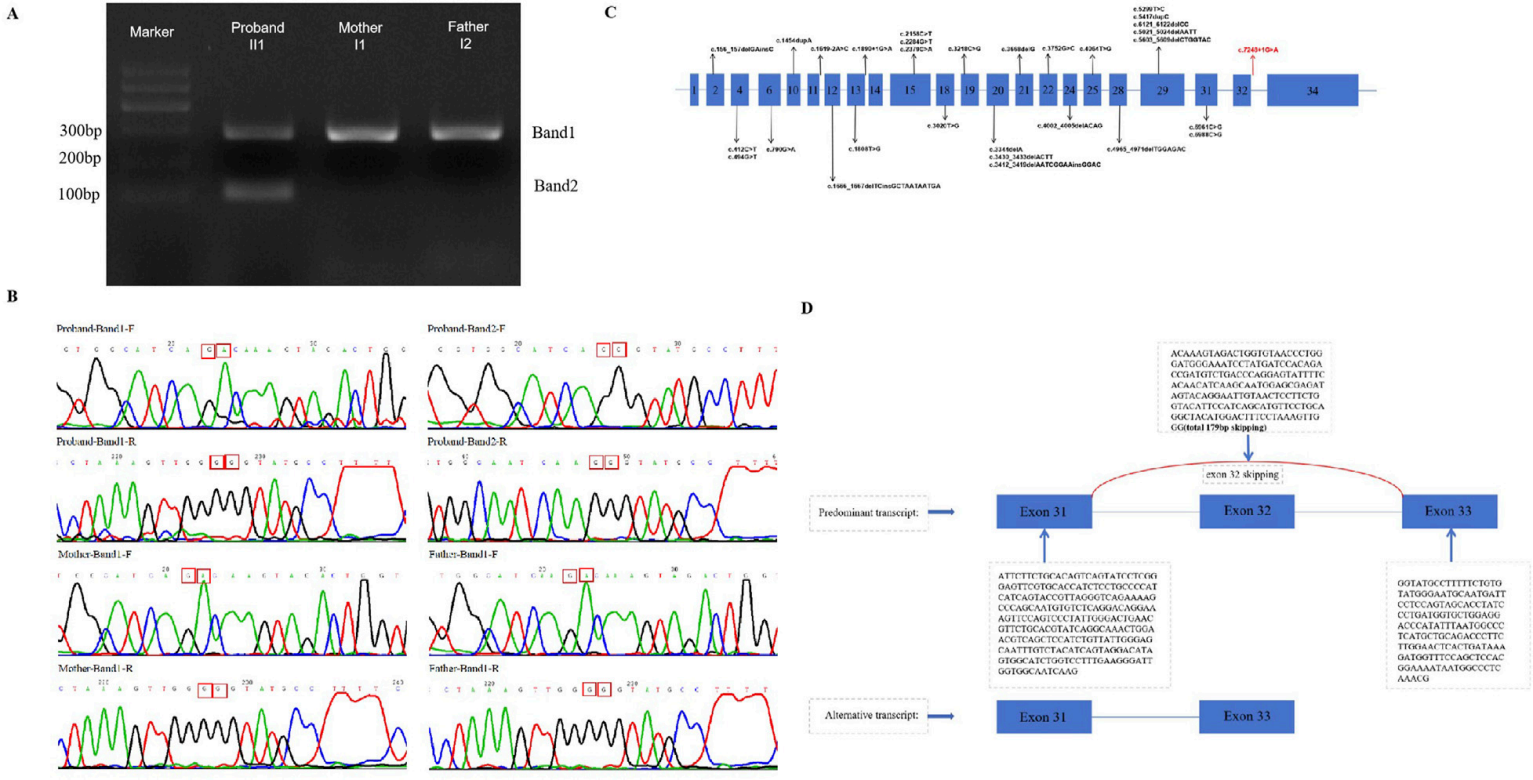
**(A)** Gel electrophoresis of the targeted cDNA fragments was performed (II1,I1,I2); **(B)** Sanger sequencing results of cDNA PCR products (Band one and 2) were obtained using forward and reverse primers, respectively; **(C)** A schematic of the *ANKRD17* gene (NM_001286771.3)is shown, indicating the reported variants and locations of all disease-causing mutations (DM) in the HGMD database (HGMD^®^ Professional 2023.3). The novel splicing variants found in this study are represented by red arrows; **(D)** The electropherograms of the cDNA fragment sequencing reveal exon 32 skipping of the *ANKRD17* gene.

The three splicing-predict software (SPIDEX: 1.977, dbscSNV ADA _SCOR: 0.999991058, dbscSNV_RF_SCORE: 0.918 and spliceAI = T|ANKRD17|0.00|0.00|0.03|0.99|7|39|7|1) predict that this variant may influence splice. Thus, cDNA from the proband and his parents was acquired and the target PCR products were sequenced. Two bands (marked as Band one and two in Figure 3A) were observed after electrophoresis for the proband, while only one band (Band 1) was observed in his parents. The size of the target band is 281 bp, which is consistent with band one in both the proband and his parents. However, only the proband exhibits a second band with an approximate size of 102 bp. Direct sequencing of these cDNA PCR products with both forward and reverse primers (Figure 3B). For the splicing variant (c.7248 + 1G>A), a 179 bp deletion of *ANKRD17* mRNA was detected (Figure 3D). Instead of the classic transcription product shared by the family, the distinct splicing products presented only in the affected proband would indicate dysfunction of the *ANKRD17* gene, leading to the development of the disease.

According to the ACMG (Richards et al., 2015) criteria, the novel splicing variant c.7248 + 1G>A within the *ANKRD17* gene is classified as “pathogenic” (PVS1, PS2_M, PM2) based on the above results. According to the PLINK software (Purcell et al., 2007), the genetic relationship between the proband and his parents was consistent, and the variation was not found in the peripheral blood of the parents, indicating that the variation was *de novo* in the proband, and the phenotype of the *ANKRD17* gene was consistent with that of the proband, but did not have specificity, so PS2 was downgraded to PS2_M. In addition, WES analysis revealed no other likely pathogen or pathogenic SNVs or CNVs associated with the proband’s clinical phenotype.

## Discussion and conclusion

Chopra-Amiel-Gordon syndrome, also known as ANKRD17-Related Neurodevelopmental Syndrome, is caused by the heterozygous loss-of-function (LOF) pathogenic variants in the *ANKRD17* gene. The *ANKRD17* gene is located on chromosome 4q13 and inherited in an autosomal dominant (AD) pathogenic pattern. The protein encoded by the *ANKRD17* gene is a member of the ankyrin repeat-containing protein family, possessing two distinct arrays of ankyrin repeats in its amino-terminal region: one with 15 ankyrin repeats and the other with 10 ankyrin repeats. Ankyrin repeats serve as protein-protein interaction modules and are associated with various biological processes. *The ANKRD17* gene has been demonstrated to contribute to DNA replication during the S-phase of the cell cycle in human cells (Deng et al., 2009; National Center for Biotechnology Information, 2023; Ghosh et al., 2012). Additionally, the *ANKRD17* gene is also likely to exert a significant influence on meiosis and the response to mismatch repair (Ray et al., 2012). The Ankyrin repeat protein *ANKRD17* interacts with Retinoic acid-inducible gene-I (RIG-I), melanoma differentiation-associated gene 5 (MDA5), and virus-induced signaling adaptor (VISA) to enhance RLR-mediated immune signaling. Overexpression or knockout of *ANKRD17* expression both impairs RLR signaling (Wang et al., 2012). The proband in our study exhibited a history of recurrent lung infections and frequent urination, potentially indicating immune impairment. However, further mechanistic investigations are required to substantiate this association in future studies.

Chopra-Amiel-Gordon syndrome is an ultra-rare disorder, characterized by developmental delay (DD), intellectual disability (ID), speech delay, behavioral disorders (spectrum disorder and ADHD), facial dysmorphism (including a triangular face, high anterior hairline, and low-set ears, etc.), and various other features (Chopra et al., 2021; Silverstein et al., 2022). The observed phenotype of the proband in our study is essentially consistent with this finding. Development and cognitive abilities are variable, with disabilities ranging from borderline to severe. The presence of normal cognition has also been documented in patients. However, almost all individuals with *ANKRD17* gene variants exhibit speech delay, including those with intellectual abilities within the normal range. The mutation spectrum of *ANKRD17*-associated neurodevelopmental syndrome, coupled with the gene’s intolerance to loss of function in the general population (gnomAD pLI score = 1), strongly suggests haploinsufficiency as the underlying disease mechanism (Sveden et al., 1993).

Only one case report suggests a potential association between the variant of the *ANKRD17* gene (c.6988A>G) and neonatal aneurysm rupture, which leads to subarachnoid hemorrhage (Silverstein et al., 2022). Qiu‐Xia Yu, et al. reported that the *de novo* missense variant of the *ANKRD17* gene (c.1525C>T) may be associated with prenatal isolated clubfoot (Yu et al., 2022). What’s more, the *ANKRD17* gene is also related to cell migration ability and plays a role in tumor metastasis, such as oral cancer and ovarian cancer,etc (Liu et al., 2014; D'Souza et al., 2017). The majority of the reported *ANKRD17* gene variants are *de novo*, and there have been no documented cases of incomplete penetrance, which is consistent with the findings presented in our article. However, since the first reported case with this splicing variation in the *ANKRD17* gene, further investigations into the underlying mechanisms are warranted in future studies.

In our study, we have confirmed that the c.7248 + 1G>A variant results in the loss of a donor splice site, leading to exon 32 skipping (r.7100_7278del179), premature termination of translation. The transcript (NM_001286771.3) of the *ANKRD17* gene consists of 34 exons, which encode a total of 2,490 amino acids. Notably, exon 32 skipping results in approximately a 2% loss of the amino acid sequences. Therefore, it can be classified as a pathogenic variant according to established ACMG (Richards et al., 2015) classification criteria. Furthermore, there is currently limited research reports on the *ANKRD17* gene. The HGMD database currently includes only two splicing variants (c.1619-2A>C and c.1890 + 1G>A) and our novel variant contributes to the overall expansion of reported *ANKRD17* gene variants (Figure 3C). Our finding expands the genotypic spectrum of *ANKRD17* gene. Furthermore, further elucidation of the specific pathogenic mechanism of this variant requires additional *in vitro* and *in vivo* experiments.

Our study suggests that the variants of the *ANKRD17* gene may be associated with partial eating, frequent urination, and tic syndrome, in addition to established phenotypes such as developmental delay. Our findings expands the phenotypic and genotypic spectrum of *ANKRD17* gene. Currently, there is no curative therapy available for ANKRD17-Related Neurodevelopmental Syndrome, however, a definitive diagnosis of its genetic etiology is significant for genetic counseling and family planning. What’s more, this is the first case report of the *ANKRD17* gene being reported in China.

## Data Availability

The variation data reported in this paper have been deposited in the Genome Variation MapGVM in National Genomies Data Center, Beijing Institute of Genomics. Chinese Academy of Sciences and China National Center for Bioinformation, under accession number GVM000811 (https://ngdc.cncb.ac.cn/gvm/).
