# Supplementary figures and images for: Case report: Whole exome sequencing reveals a novel splicing variant of ANKRD17 gene in a Chinese male juvenile with developmental delay and transient tic disorder
Source: Front Genet. 2024 Sep 9;15:1422469. doi: 10.3389/fgene.2024.1422469 (PMC11416919; doi:10.3389/fgene.2024.1422469)

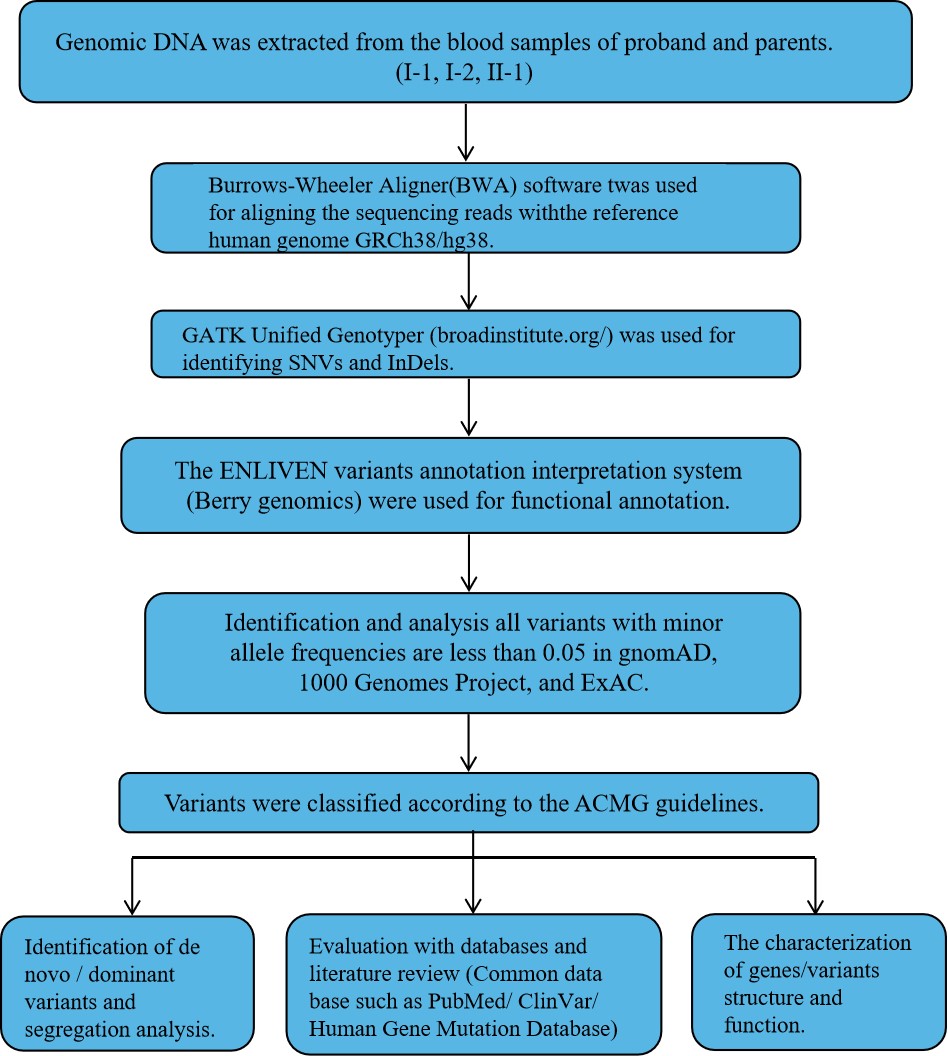

Supplement: Supplementary file 1 [file Image1.JPEG]
